# Supplementary material for: Specific DNMT3C flanking sequence preferences facilitate methylation of young murine retrotransposons
Source: Commun Biol. 2024 May 16;7:582. doi: 10.1038/s42003-024-06252-z (PMC11099192; doi:10.1038/s42003-024-06252-z)
Supplement: Supplementary file 2 — Supplementary information [file 42003_2024_6252_MOESM2_ESM.pdf]

Specific DNMT3C flanking sequence preferences facilitate methylation of young murine retrotransposons

Leonie Dossmann, Max Emperle, Michael Dukatz, Alex de Mendozza, Pavel Bashtrykov & Albert Jeltsch

Supplementary information

Supplementary Figures

Supplementary Fig. 1 Sequence alignment of catalytic domains of human and murine DNMT3A and DNMT3B with DNMT3C.

Supplementary Fig. 2 Correlation of the -8 to +8 flanking profiles of all individual data sets.

Supplementary Fig. 3 Combined -8 to +8 flanking profiles of all individual data sets.

Supplementary Fig. 4 Methylation rates of DNA substrates in hm and fm form.

Supplementary Fig. 5 Examples of the exponential curve fitting of methylation rates of different NNCGNN substrates.

Supplementary Fig. 6 Non-CpG activity of DNMT3C.

Supplementary Fig. 7 Flanking sequence preferences of DNMT3C V547I and V547G resemble DNMT3C

Supplementary Fig. 8 DNA methylation in 6KO and 7KO cells.

Supplementary Fig. 9 Example images of local correlation of methylation levels and DNMT3C NNCGNN preferences for consecutive CpG sites in chromosome 1 of 6KO cells.

Supplementary Fig. 10 Promoter sequences of L1 elements strongly methylated by DNMT3C.
Supplementary Fig. 11 Examples of transcription factor binding site motifs containing CCCG or CGGG motifs.

Supplementary Tables

Supplementary Table 1 Sequencing statistics for the methylation experiments of single CpG site substrates in randomized sequence context including DNMT3C concentrations, incubation times and methylation levels.

Supplementary Table 2 Sequencing statistics for the methylation experiments of single CpX site substrates in randomized sequence context including DNMT3C concentrations, incubation times and methylation levels.

Supplementary Table 3 Sequences of the methylation substrates used. CpG sites are marked in red.

Supplementary Table 4 Genomic coordinates of the CpG sites shown in Supplementary Fig. 8.

Supplementary Table 5 CpG frequency and frequency of CG sites in a CCCG or CGGG context in different repeat elements.

Supplementary references

Supplementary Figures

Supplementary Fig. 1 Sequence alignment of catalytic domains of human and murine DNMT3A and DNMT3B with DNMT3C. C543, V547, and E590 in DNMT3C are shaded red. The arrow highlights the start of the cloned catalytic domain.

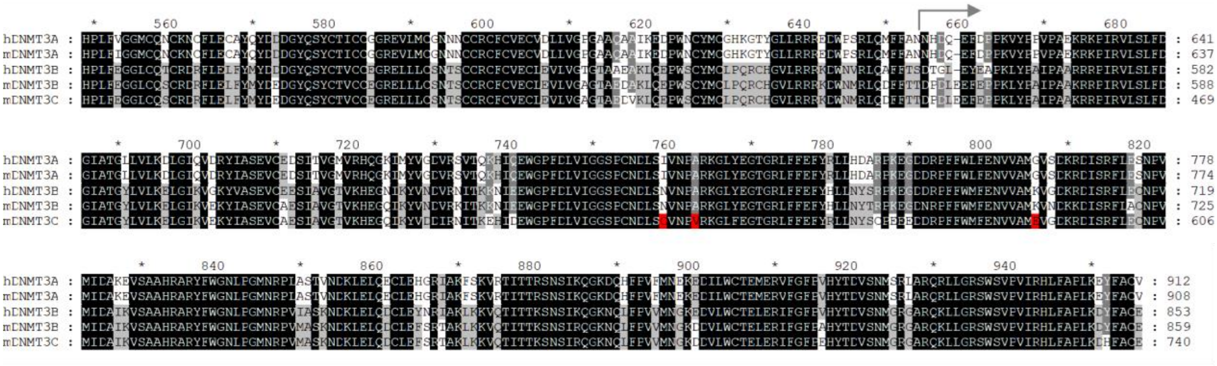

Supplementary Fig. 2 Correlation of the -8 to +8 flanking profiles of all individual data sets. Shown are Pearson r-values.

			DNMT3C													DNMT3C C543N/V547A						DNMT3C V547I		DNMT3C V547G	
			CpG							CpN						CpG			CpN			CpG		CpG	
			R1	R2	R3	R4	R5	R6	R7	R1	R2	R3	R4	R5	R6	R1	R2	R3	R1	R2	R3	R1	R2	R1	R2
DNMT3C	CpG	R1	1.00	0.94	0.95	0.96	0.93	0.94	0.94	0.94	0.92	0.95	0.95	0.93	0.94	0.85	0.87	0.85	0.86	0.86	0.88	0.88	0.92	0.87	0.91
		R2	0.94	1.00	0.96	0.96	0.95	0.96	0.95	0.96	0.93	0.95	0.96	0.96	0.92	0.86	0.87	0.86	0.87	0.87	0.88	0.91	0.93	0.91	0.93
		R3	0.95	0.96	1.00	0.99	0.96	0.97	0.97	0.98	0.95	0.96	0.97	0.96	0.94	0.87	0.88	0.87	0.87	0.88	0.89	0.94	0.94	0.90	0.95
		R4	0.96	0.96	0.99	1.00	0.97	0.98	0.97	0.97	0.96	0.97	0.97	0.96	0.95	0.89	0.90	0.89	0.89	0.90	0.90	0.93	0.93	0.91	0.95
		R5	0.93	0.95	0.96	0.97	1.00	0.99	0.98	0.95	0.95	0.95	0.96	0.98	0.91	0.86	0.87	0.87	0.86	0.86	0.87	0.95	0.96	0.94	0.96
		R6	0.94	0.96	0.97	0.98	0.99	1.00	0.99	0.96	0.95	0.96	0.97	0.98	0.93	0.86	0.87	0.87	0.86	0.87	0.88	0.96	0.97	0.95	0.97
		R7	0.94	0.95	0.97	0.97	0.98	0.99	1.00	0.95	0.95	0.94	0.97	0.97	0.93	0.85	0.86	0.86	0.85	0.86	0.87	0.95	0.96	0.93	0.96
	CpN	R1	0.94	0.96	0.98	0.97	0.95	0.96	0.95	1.00	0.93	0.94	0.95	0.95	0.92	0.86	0.87	0.87	0.86	0.86	0.88	0.87	0.90	0.85	0.90
		R2	0.92	0.93	0.95	0.96	0.95	0.95	0.95	0.93	1.00	0.94	0.94	0.94	0.93	0.87	0.88	0.88	0.88	0.88	0.89	0.92	0.93	0.89	0.93
		R3	0.95	0.95	0.96	0.97	0.95	0.96	0.94	0.94	0.94	1.00	0.97	0.95	0.93	0.87	0.88	0.87	0.87	0.88	0.89	0.92	0.92	0.88	0.93
		R4	0.95	0.96	0.97	0.97	0.96	0.97	0.97	0.95	0.94	0.97	1.00	0.97	0.93	0.86	0.87	0.86	0.86	0.87	0.88	0.90	0.92	0.90	0.93
		R5	0.93	0.96	0.96	0.96	0.98	0.98	0.97	0.95	0.94	0.95	0.97	1.00	0.90	0.84	0.85	0.84	0.84	0.84	0.86	0.93	0.95	0.92	0.95
		R6	0.94	0.92	0.94	0.95	0.91	0.93	0.93	0.92	0.93	0.93	0.93	0.90	1.00	0.85	0.86	0.85	0.86	0.86	0.87	0.93	0.96	0.92	0.96
DNMT3C C543N/V547A	CpG	R1	0.85	0.86	0.87	0.89	0.86	0.86	0.85	0.86	0.87	0.87	0.86	0.84	0.85	1.00	1.00	1.00	0.99	0.99	0.99	0.85	0.82	0.82	0.84
		R2	0.87	0.87	0.88	0.90	0.87	0.87	0.86	0.87	0.88	0.88	0.87	0.85	0.86	1.00	1.00	1.00	1.00	0.99	0.99	0.84	0.83	0.82	0.84
		R3	0.85	0.86	0.87	0.89	0.87	0.87	0.86	0.87	0.88	0.87	0.86	0.84	0.85	1.00	1.00	1.00	0.99	0.99	0.99	0.85	0.82	0.83	0.84
	CpN	R1	0.86	0.87	0.87	0.89	0.86	0.86	0.85	0.86	0.88	0.87	0.86	0.84	0.86	0.99	1.00	0.99	1.00	0.99	0.99	0.84	0.82	0.82	0.84
		R2	0.86	0.87	0.88	0.90	0.86	0.87	0.86	0.86	0.88	0.88	0.87	0.84	0.86	0.99	0.99	0.99	0.99	1.00	0.99	0.85	0.82	0.83	0.85
		R3	0.88	0.88	0.89	0.90	0.87	0.88	0.87	0.88	0.89	0.89	0.88	0.86	0.87	0.99	0.99	0.99	0.99	0.99	1.00	0.86	0.84	0.84	0.86
DNMT3C V547I	CpG	R1	0.88	0.91	0.94	0.93	0.95	0.96	0.95	0.87	0.92	0.92	0.90	0.93	0.93	0.85	0.84	0.85	0.84	0.85	0.86	1.00	0.95	0.95	0.96
		R2	0.92	0.93	0.94	0.93	0.96	0.97	0.96	0.90	0.93	0.92	0.92	0.95	0.96	0.82	0.83	0.82	0.82	0.82	0.84	0.95	1.00	0.94	0.97
DNMT3C V547G	CpG	R1	0.87	0.91	0.90	0.91	0.94	0.95	0.93	0.85	0.89	0.88	0.90	0.92	0.92	0.82	0.82	0.83	0.82	0.83	0.84	0.95	0.94	1.00	0.94
		R2	0.91	0.93	0.95	0.95	0.96	0.97	0.96	0.90	0.93	0.93	0.93	0.95	0.96	0.84	0.84	0.84	0.84	0.85	0.86	0.96	0.97	0.94	1.00

Supplementary Fig. 3 Combined -8 to +8 flanking profiles of all individual data sets. a-d Observed/expected (o/e) ratios for the occurrence of individual bases at each position in the methylated products determined for WT DNMT3C, WT DNMT3B, WT DNMT3A and the DNMT3C C543N/V547A mutant. DNMT3A data were combined from [1,2]; DNMT3B data were taken from [1].

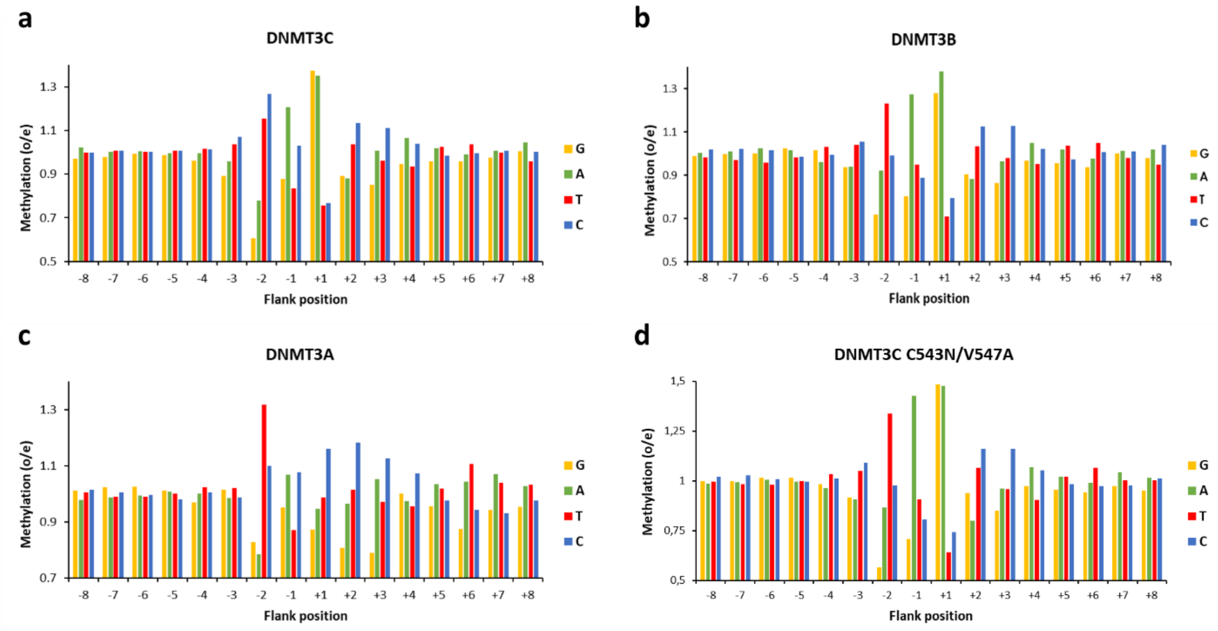

Supplementary Fig. 4 Methylation rates of DNA substrates in hm and fm form. a Exemplary methylation kinetics of the designed substrates to be preferred by DNMT3C or DNMT3B, conducted with WT DNMT3C, WT DNMT3B and DNMT3C C543N/V547A. **b** Exemplary methylation kinetics of the repeat 2 substrates with WT DNMT3C, WT DNMT3B and DNMT3C C543N/V547A mutant. For activity normalization, methylation of the reference substrate containing 38 CpG sites with equal average preferences of DNMT3C and DNMT3B is shown as well. Lines show linear regressions through the data points.

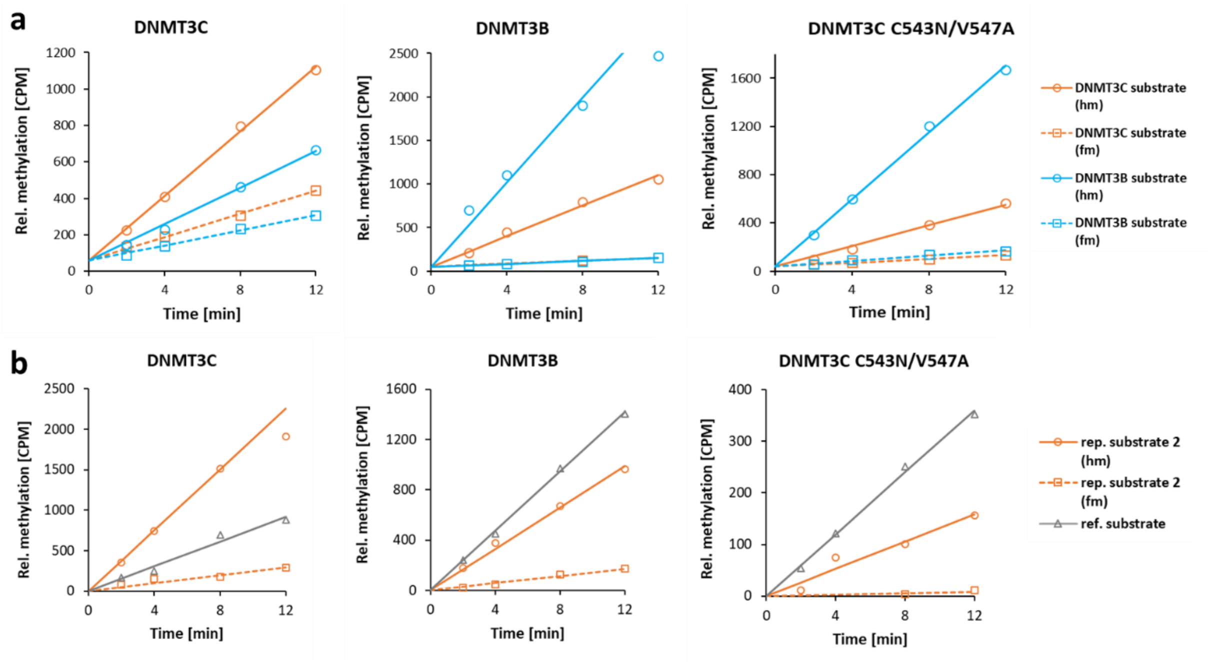

Supplementary Fig. 5 Examples for the exponential curve fitting of methylation rates of different NNCGNN substrates. In the fitting of WT DNMT3C and DNMT3C C543N/V547A mutant a virtual time scale (arbitrary units, a.u.) is used to relate the data set to one another. The lines show the fits to the data.

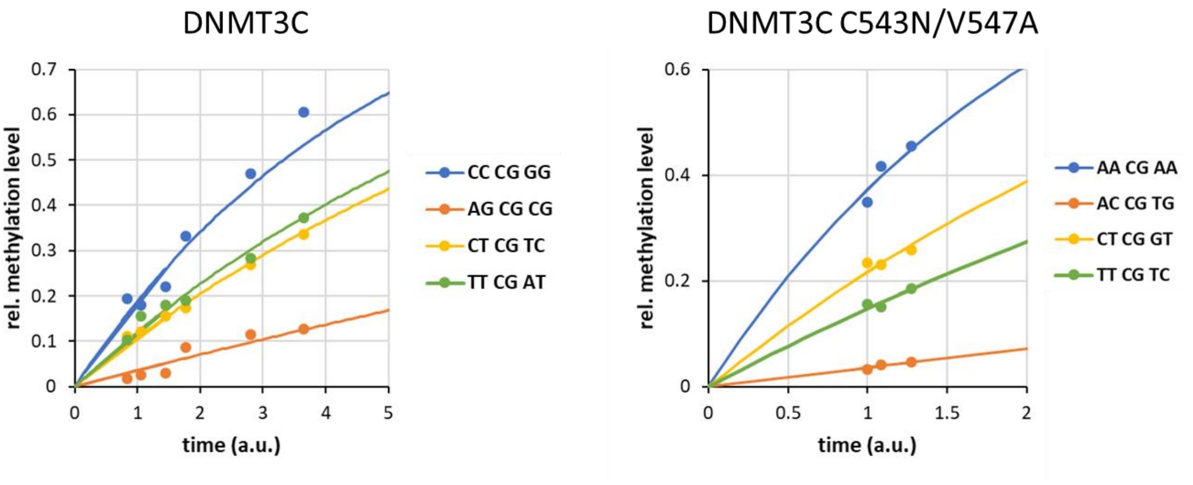

Supplementary Fig. 6 Non-CpG activity of DNMT3C. a Exponential fit of the methylation of CpG and non-CpG substrates for DNMT3C and DNMT3B. DNMT3B data were taken from [3]. The lines show the fit to the data. **b** Comparison of relative non-CpG methylation activities of DNMT3C and DNMT3B. **c)** Enrichment and depletion of bases at the -2 to +2 flank of the 10% most preferred NNCXNN sequences for CpG, CpA and CpT methylation by WT DNMT3C.

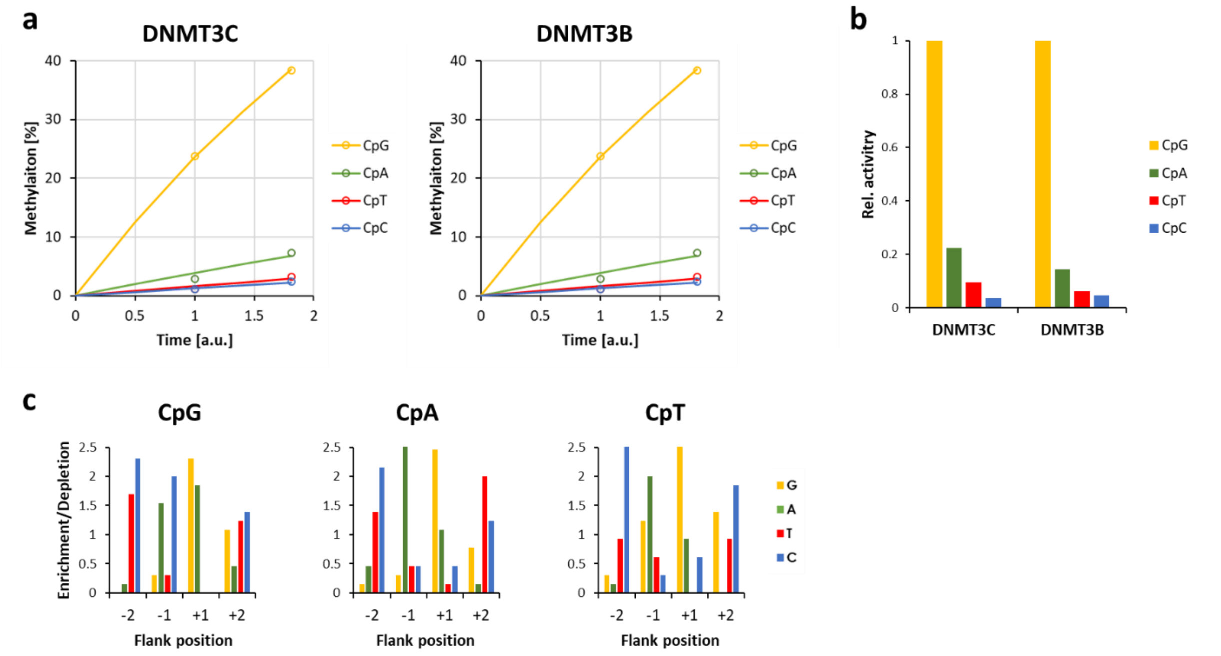

Supplementary Fig. 7 Flanking sequence preferences of DNMT3C V547I and V547G mutants resemble DNMT3C. a o/e ratios for the occurrence of individual bases at the -4 to +4 position in the methylated products determined for WT DNMT3C, WT DNMT3B, DNMT3C V547I, and DNMT3C V547G. DNMT3B data were taken from [1]. The C-preference at the -2 and -1 sites that is specific for DNMT3C is highlighted by blue arrows. **b** Pearson r-values for the correlation of the -8 to +8 flanking profiles of WT DNMT3C, DNMT3C V547I, DNMT3C V547G and WT DNMT3B.

a

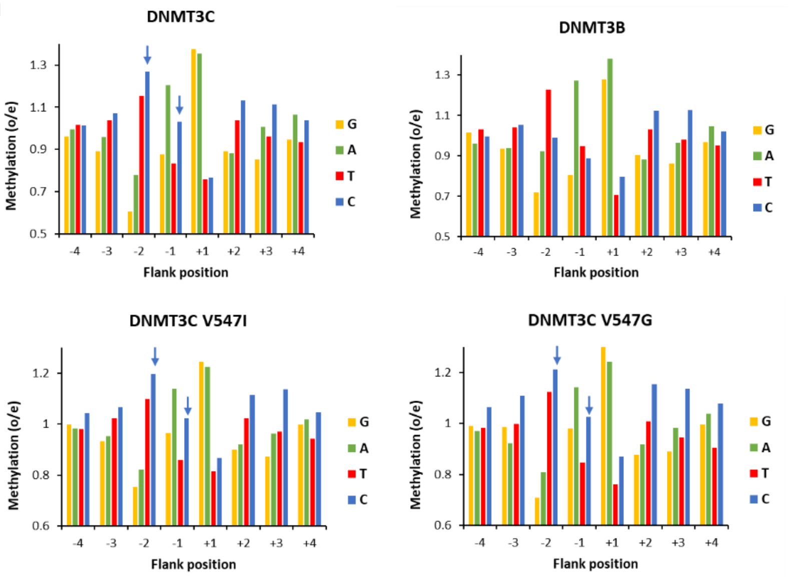

b

	DNMT3C	DNMT3C V547I	DNMT3C V547G	DNMT3B
DNMT3C	1.00	0.96	0.94	0.88
DNMT3C V547I	0.96	1.00	0.98	0.84
DNMT3C V547G	0.94	0.98	1.00	0.83
DNMT3B	0.88	0.84	0.83	1.00

Supplementary Fig. 8 DNA methylation in 6KO and 7KO cells [4]. a Genetic background of the 6KO and 7KO cells is indicated. **b** Box plot of the average methylation in NNCGNN sequences. The box shows the median, 1st and 3rd quartile. Whiskers display the 1.5 IQR distance. Points represent outliers.

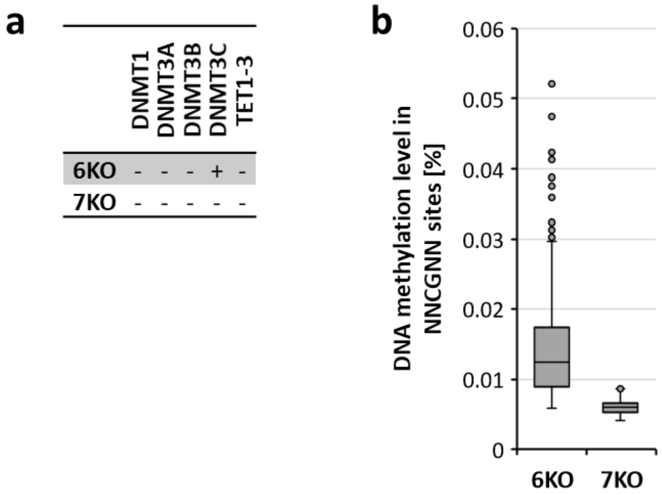

Supplementary Fig. 9 Example images of local correlation of methylation levels and DNMT3C NNCGNN preferences for consecutive CpG sites in chromosome 1 of 6KO cells. a-f The individual images show 32 consecutive CpG sites. Relative preferences were scaled to fit the methylation levels. The coordinates of the CpG sites are given in Supplementary Table 4.

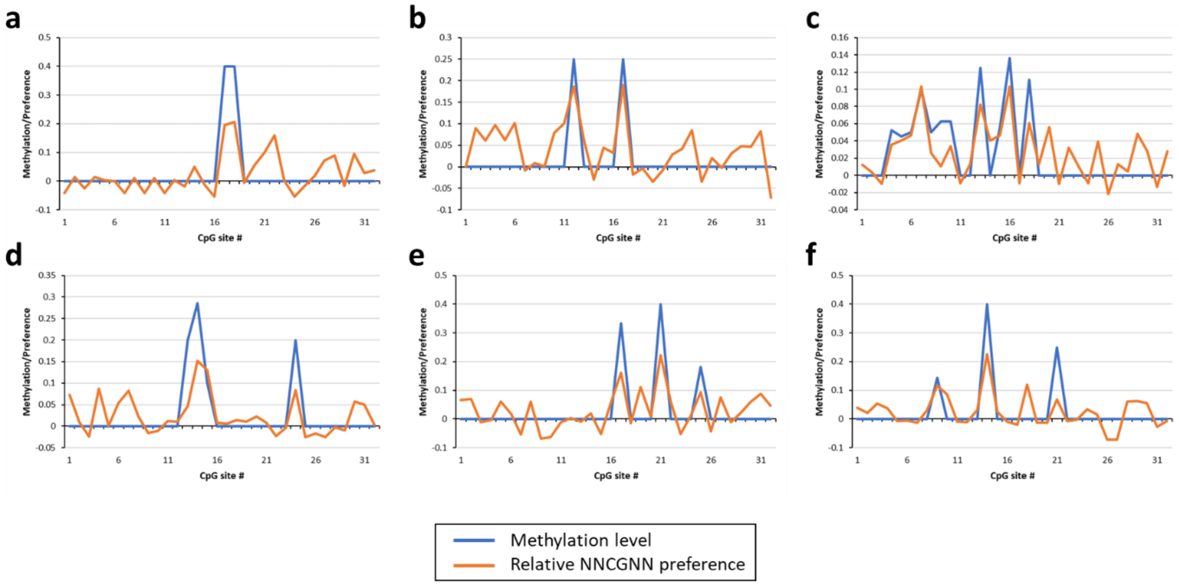

Supplementary Fig. 10 Promoter sequences of L1 elements strongly methylated by DNMT3C. CpG sites are shaded in yellow, DNMT3C preferred CCCG and CGGG sites in green. Sequences promoter information were taken from the Dfam database [5].

L1Md_A Promoter, DF000001807

CGACTCGAGACTCGAGCCCCGGGCTACCTTGCCAGCAGAGTCTTGCCCAACACCCGCAAGGGTCCACACGGGA
CTCCCCACGGGACCCTAAGACCTCTGGTGAGTGGATCACAGTGCCTGCCCCAATCCAATCGCGCGGAACTTGA
GACTGCGGTACATAGGGAAGCAGGCTACCCGGGCCTGATCTGGGGCACAAGTCCCTTCCGCTCGACTCGAGA
CTCGAGCCCCGGGCTACCTTGCCAGCAGAGTCTTGCCCAACACCCGCAAGGGTCCACACGGGACTCCCCACGG
GACCCTAAGACCTCTGGTGAGTGGATCACAGTGCCTGCCCCAATCCAATCGCGCGGAACTTGAGACTGCGGTA
CATAGGGAAGCAGGCTACCCGGGCCTGATCTGGGGCACAAGTCCCTTCCGCTCGACTCGAGACTCGAGCCCCG
GGCTACCTTGCCAGCAGAGTCTTGCCCAACACCCGCAAGGGCCCACACGGGACTCCCCACGGGACCCTAAGAC
CTCTGGTGAGTGGANCACAGCGCCTGCCCCAATCCAATCGCGCGGAACTTGAGACTGCGGTACATAGGGAAG
CAGGCTACCCGGGCTTGATCTGGGGCACAAACCCCTTCCGCTCCACTCGAGCCCCGGCTACCTTGCCAGCTGA
GTCGCCCGACACCCGCAAGGGCCCACACGGGATTCCACACGTGATCCTAAGACCTCTAGTGAGTG

L1Md_T Promoter, DF000001864

TGTGGGCCGGGGACAGCCGGCCACCTTCCGGACCGGAGGACAGGTGCCCGCCCGGCTGGGGAGGCGACCTA
AGCCACAGCAGCAGCGGTCGCCATCTTGGTCCGGGACCCGCCGAACTTAGGAAATTAGTCTGAACAGGTGAG
AGGGTGCGCCAGAGAACCTGACAGCTTCTGGAACAGGCGGAAGCACAGAGGCGCTGAGGCAGCACCCTGTG
TGGGCCGGGGACAGCCGGCCACCTTCCGGACCGGAGGACAGGTGCCCGCCCGGCTGGGGAGGCGGCCTAAG
CCACAGCAGCAGCGGTCGCCATCTTGGTCCGGGACCCGCCGAACTTAGGAAATTAGTCTGAACAGGTGAGAG
GGTGCGCCAGAGAACCTGACAGCTTCTGGAACAGGCGGAAGCACAGAGGCGCTGAGGCAGCACCCTGTGTG
GGCCGGGGACAGCCGGCCACCTTCCGGACCGGAGGACAGGTGCCCGCCCGGCTGGGGAGGCGACCTAAGCC
ACAGCAGCAGCGGTCGCCATCTTGGTCCCGGGA

L1Md_Gf Promoter, DF000001849

GAGAGCACGGGGGTCTGCCTGGCCTGAGAGGTTTGTGGCACAGGCGCCGGCGGGAGCCTTCTTGGCTCCGG
GACTCCGCGGAGGGCAGGCTGCACGGGTGACCGTGTGGAATACAGAGTGCCAGCCGTTTCTGGGACGGGCG
AGAGAGTCGCAGAGCTTCTGGGCGGCGCCATCTTCAGCTCCAGACAGCCGGCCACCTTCCTGGTGAGAGCAC
GGGGGTCTGCCTGGCCTGAGAGGTTTGTGGCACAGGCGCCGGCGGGAGCCTTCTTGGCTCCGGGACTCCGCG
GAGGGCAGGCTGCACGGGTGACCGTGTGGAATACAGAGTGCCAGCCGTTTCTGGGACGGGCGAGAGAGTCG
CAGAGCTTCTGGGGCGGCGCCATCCTCAGCTCCAGACGGCCGGCCACCTTCCCGG

Supplementary Fig. 11 Examples of transcription factor binding site motifs containing CCCG or CGGG motifs. Images were taken from the Jaspar data base [6].

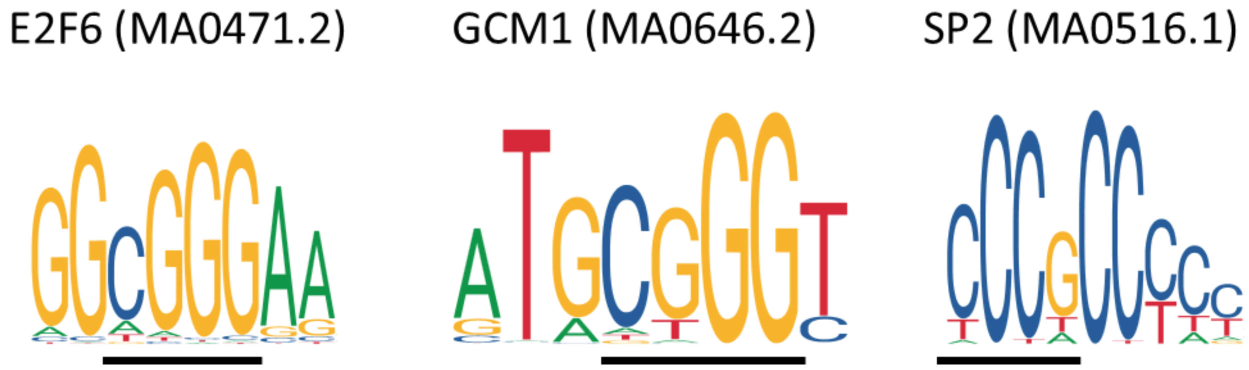

Supplementary Tables

Supplementary Table 1 Sequencing statistics for the methylation experiments of single CpG site substrates in randomized sequence context including DNMT3C concentrations, incubation times and methylation levels.

Experiment		c(DNMT3) [µM]	Incubation time [min]	CG substrate		
				met	unmet	%met
DNMT3C	R1	1.2	30	2388	40285	5.60
	R2		60	6330	64234	8.97
	R3	4.9	30	11883	98956	10.72
	R4		60	13838	82177	14.41
	R5	9.8		15074	73427	17.03
	R6	14.7		18621	52953	26.02
	R7	19.2		14727	31343	31.97
DNMT3C C543N/V547A	R1	10.4	60	57705	238110	19.51
	R2	15.6		50107	172832	22.48
	R3	20.8		48070	212358	18.46
DNMT3C V547I	R1	11.2	60	4940	8829	35.88
	R2	22.4		14908	19354	43.51
DNMT3C V547G	R1	6.4		3491	14478	19.43
	R2	12.8		4502	6242	41.90

Supplementary Table 2 Sequencing statistics for the methylation experiments of single CpX site substrates in randomized sequence context including DNMT3C concentrations, incubation times and methylation levels.

Experiment		c(DNMT3) [µM]	Incuba-tion time [min]	CN substrate (CpG)			CN substrate (CpA)			CN substrate (CpT)			CN substrate (CpC)		
				met	unmet	%met	met	unmet	%met	met	unmet	%met	met	unmet	%met
DNMT3C	R1	1.2	60	2317	18333	11.22	504	16894	2.90	228	16781	1.34	90	16257	0.55
	R2	4.9	30	2966	16649	15.12	450	16976	2.58	218	17301	1.24	84	16522	0.51
	R3		60	2744	17091	13.83	604	16984	3.43	231	16888	1.35	100	16246	0.61
	R4	9.8		5084	15745	24.41	1590	21980	6.75	576	19831	2.82	199	18966	1.04
	R5	14.7		5969	15995	27.18	1847	22792	7.50	715	20486	3.37	222	19480	1.13
	R6	19.6		4267	10880	28.17	1216	17932	6.35	504	18031	2.72	198	15755	1.24
DNMT3C C543N/V547A	R1	10.4	60	14543	46299	23.90	1627	39414	3.96	591	39244	1.48	362	34074	1.05
	R2	15.6		16518	60824	21.36	2028	52977	3.69	817	52914	1.52	488	44784	1.08
	R3	20.8		14197	52764	21.20	1698	44208	3.70	674	44683	1.49	440	39074	1.11

Supplementary Table 3 Sequences of the methylation substrates used. CpG sites are marked in red.

Substrate	Sequence
**CG-rich**	GGTCCTCTTTTCCTCCTCTCCAGCTCCGGCGCCGTAGCCATCATGGTGAGTCTCCTCG<br>GGCTCTCAGCACTATCCGTCGCTTATCCGCGCCATCCCTGCCTGTTCGGGGACCCCAC<br>TAGAGCTGCAGCCATCCGGGGCCCACTTACTCTGCTCCGGCCTGCCTCACGGAGGCC<br>GGTCGCCCGGGGTCGCCGAGTCGCGGCTGTCCGGCACCGCCGTCCGGAACACGCCG<br>GGCTTTGGGCTCCCGGGCCTCGGCTATGGAAACCCCTGTGGAGCTTCAGGGGCACG<br>AGTGAGGCGGGCGCTGGCGGGCCAAGGTGACGAAGGCGCCTCCGGCTCTTGGGCC<br>AGCGGACTGAGCGGTGGAGCAGAACTTGGGTGCCTCGGGGACCGCCAAAAAGTGG<br>CCTTGTCCACTTCTCTGAG
**DNMT3B-preferred**	GAAGCTGGGAATTACGACAGGAGAGTGCAA
**DNMT3C-preferred**	GAAGCTGGGAATCCCGACAGGAGAGTGCAA
**Repeat 1**	GGAAGCAGGCTACCCGGGCCTGATCTGGGG
**Repeat 2**	GAAGCTGGGTGGTCCGGGACCAGAGTGCAA

Supplementary Table 4 Genomic coordinates of the CpG sites shown in Supplementary Fig. 8.

		Panel					
		A	B	C	D	E	F
CpG site #	1	chr1(77972528-77972550)	chr1(41073562-41073584)	chr1(33076404-33076426)	chr1(84281231-84281253)	chr1(188162075-188162097)	chr1(50932142-50932164)
	2	chr1(77972559-77972581)	chr1(41073615-41073637)	chr1(33076408-33076430)	chr1(84281239-84281261)	chr1(188162110-188162132)	chr1(50932681-50932703)
	3	chr1(77972575-77972597)	chr1(41073659-41073681)	chr1(33076429-33076451)	chr1(84281241-84281263)	chr1(188169607-188169629)	chr1(50932712-50932734)
	4	chr1(77972606-77972628)	chr1(41073665-41073687)	chr1(33076441-33076463)	chr1(84281418-84281440)	chr1(188170134-188170156)	chr1(50938423-50938445)
	5	chr1(77972620-77972642)	chr1(41073675-41073697)	chr1(33076465-33076487)	chr1(84281727-84281749)	chr1(188170249-188170271)	chr1(50938428-50938450)
	6	chr1(77972622-77972644)	chr1(41073687-41073709)	chr1(33076476-33076498)	chr1(84281991-84282013)	chr1(188170255-188170277)	chr1(50938477-50938499)
	7	chr1(77972628-77972650)	chr1(41073689-41073711)	chr1(33076486-33076508)	chr1(84282082-84282104)	chr1(188170265-188170287)	chr1(50938503-50938525)
	8	chr1(77972675-77972697)	chr1(41073692-41073714)	chr1(33076493-33076515)	chr1(84282098-84282120)	chr1(188170826-188170848)	chr1(50938520-50938542)
	9	chr1(77972681-77972703)	chr1(41073710-41073732)	chr1(33076511-33076533)	chr1(84282211-84282233)	chr1(188172625-188172647)	chr1(50938536-50938558)
	10	chr1(77972728-77972750)	chr1(41073716-41073738)	chr1(33076525-33076547)	chr1(84285150-84285172)	chr1(188172823-188172845)	chr1(50938543-50938565)
	11	chr1(77972734-77972756)	chr1(41073721-41073743)	chr1(33076556-33076578)	chr1(84287193-84287215)	chr1(188172880-188172902)	chr1(50938552-50938574)
	12	chr1(77972779-77972801)	chr1(41074774-41074796)	chr1(33076656-33076678)	chr1(84287274-84287296)	chr1(188173022-188173044)	chr1(50951608-50951630)
	13	chr1(77972781-77972803)	chr1(41075024-41075046)	chr1(33076671-33076693)	chr1(84290144-84290166)	chr1(188174426-188174448)	chr1(50957231-50957253)
	14	chr1(77972795-77972817)	chr1(41075140-41075162)	chr1(33076676-33076698)	chr1(84290156-84290178)	chr1(188174570-188174592)	chr1(50957544-50957566)
	15	chr1(77972890-77972912)	chr1(41077877-41077899)	chr1(33076687-33076709)	chr1(84290219-84290241)	chr1(188178977-188178999)	chr1(50971152-50971174)
	16	chr1(77972907-77972929)	chr1(41077885-41077907)	chr1(33076697-33076719)	chr1(84290293-84290315)	chr1(188181684-188181706)	chr1(50982487-50982509)
	17	chr1(77972939-77972961)	chr1(41077888-41077910)	chr1(33076767-33076789)	chr1(84290345-84290367)	chr1(188182102-188182124)	chr1(50982515-50982537)
	18	chr1(77974992-77975014)	chr1(41079658-41079680)	chr1(33076804-33076826)	chr1(84290932-84290954)	chr1(188182119-188182141)	chr1(50982527-50982549)
	19	chr1(77975782-77975804)	chr1(41081514-41081536)	chr1(33076807-33076829)	chr1(84292018-84292040)	chr1(188182547-188182569)	chr1(50982585-50982607)
	20	chr1(77979143-77979165)	chr1(41085223-41085245)	chr1(33077904-33077926)	chr1(84292051-84292073)	chr1(188185688-188185710)	chr1(50982619-50982641)
	21	chr1(77979187-77979209)	chr1(41085235-41085257)	chr1(33078295-33078317)	chr1(84292766-84292788)	chr1(188193816-188193838)	chr1(50982702-50982724)
	22	chr1(77981703-77981725)	chr1(41085978-41086000)	chr1(33078314-33078336)	chr1(84302143-84302165)	chr1(188196815-188196837)	chr1(50982715-50982737)
	23	chr1(77981767-77981789)	chr1(41086964-41086986)	chr1(33078407-33078429)	chr1(84302159-84302181)	chr1(188196903-188196925)	chr1(50982806-50982828)
	24	chr1(77981793-77981815)	chr1(41087013-41087035)	chr1(33078418-33078440)	chr1(84302831-84302853)	chr1(188198489-188198511)	chr1(50982826-50982848)
	25	chr1(77981798-77981820)	chr1(41087402-41087424)	chr1(33079598-33079620)	chr1(84303072-84303094)	chr1(188198514-188198536)	chr1(50982939-50982961)
	26	chr1(77981800-77981822)	chr1(41087427-41087449)	chr1(33080458-33080480)	chr1(84303081-84303103)	chr1(188198549-188198571)	chr1(50983386-50983408)
	27	chr1(77981841-77981863)	chr1(41090707-41090729)	chr1(33080481-33080503)	chr1(84303268-84303290)	chr1(188207873-188207895)	chr1(50983399-50983421)
	28	chr1(77981849-77981871)	chr1(41094177-41094199)	chr1(33093693-33093715)	chr1(84303271-84303293)	chr1(188210278-188210300)	chr1(50983424-50983446)
	29	chr1(77981864-77981886)	chr1(41094187-41094209)	chr1(33099282-33099304)	chr1(84303325-84303347)	chr1(188212149-188212171)	chr1(50983432-50983454)
	30	chr1(77983478-77983500)	chr1(41094195-41094217)	chr1(33102694-33102716)	chr1(84304363-84304385)	chr1(188212206-188212228)	chr1(50983490-50983512)
	31	chr1(77983839-77983861)	chr1(41094214-41094236)	chr1(33119119-33119141)	chr1(84307661-84307683)	chr1(188215301-188215323)	chr1(50983501-50983523)
	32	chr1(77983857-77983879)	chr1(41094243-41094265)	chr1(33120952-33120974)	chr1(84312755-84312777)	chr1(188216312-188216334)	chr1(50983578-50983600)

Supplementary Table 5 CpG frequency and frequency of CG sites in a CCCG or CGGG context in different repeat elements. In the case of the L1Md_A, L1Md_T and L1Md_Gf, promoter regions with clustered CpG sites were used. The sequences are given in Supplementary Fig. 10. For all other repeat elements, the entire DFAM entry was used for analysis. For the calculation of expected frequencies, the nucleotide composition of the corresponding repeat element was used.

Name	DFAM	Repeat class	DNMT3C target	Length	CpG		CCCG+CGGG	
					Number	o/e	Number	o/e
L1Md_A promoter	DF0001807	Line	+	720	47	0.89	25	3.52
L1Md_T promoter	DF0001864	LINE	+	532	40	1.03	15	2.56
L1Md_Gf promoter	DF0001849	LINE	+	411	34	1.17	11	2.28
MMERVK10C	DF000001942.1	ERVK	+	7581	151	0.45	36	2.65
IAPEz-int	DF0004146	ERVK	+	6481	153	0.45	37	2.30
IAP_d	DF0001776	ERVK	+	6542	162	0.46	56	3.17
MMERVK9E	DF000001946.1	ERVK	-	6152	154	0.53	26	1.76
MMERVK10D3	DF000001943.2	ERVK	-	6156	177	0.54	35	1.79
MMVL30	DF000004184.1	ERV1	-	3698	107	0.51	12	0.98
MERVL	DF000003918.1	ERVL	-	5485	73	0.27	8	1.08
HERV (Primate)	DF000000188.5	ERV2	-	7536	156	0.47	23	1.67

Supplementary references

1 Gao, L. *et al.* Comprehensive structure-function characterization of DNMT3B and DNMT3A reveals distinctive de novo DNA methylation mechanisms. *Nature communications* **11**, 3355 (2020).

2 Dukatz, M. *et al.* DNA methyltransferase DNMT3A forms interaction networks with the CpG site and flanking sequence elements for efficient methylation. *J Biol Chem* **298**, 102462 (2022).

3 Dukatz, M. *et al.* Complex DNA sequence readout mechanisms of the DNMT3B DNA methyltransferase. *Nucleic Acids Res* **48**, 11495-11509 (2020).

4 Wang, Q. *et al.* Imprecise DNMT1 activity coupled with neighbor-guided correction enables robust yet flexible epigenetic inheritance. *Nat Genet* **52**, 828-839 (2020).

5 Hubley, R. *et al.* The Dfam database of repetitive DNA families. *Nucleic Acids Res* **44**, D81-89 (2016).

6 Rauluseviciute, I. *et al.* JASPAR 2024: 20th anniversary of the open-access database of transcription factor binding profiles. *Nucleic Acids Res* (2023).
