# Supplementary material for: Specific DNMT3C flanking sequence preferences facilitate methylation of young murine retrotransposons
Source: Commun Biol. 2024 May 16;7:582. doi: 10.1038/s42003-024-06252-z (PMC11099192; doi:10.1038/s42003-024-06252-z)
Supplement: Supplementary file 3 — Description of additional supplementary files [file 42003_2024_6252_MOESM3_ESM.pdf]

Description of Additional Supplementary Files

File name: Supplementary Data 1

Description: Source data and uncropped imaged of all figures.
